# What predicts continued substance use among probationers?

**DOI:** 10.1186/1940-0640-10-S1-A33

**Published:** 2015-02-20

**Authors:** Jennifer Lerch, Scott T Walters, Faye S Taxman

**Affiliations:** 1 Criminology, Law & Society, George Mason University, Fairfax, VA, 22030, USA; 2 Behavioral and Community Health, University of North Texas Health Science Center, Fort Worth, TX, 76107, USA

## Background

Because of the strong connection between substance use and criminality, one primary aim of probation is to suppress substance use as a way to reduce criminal behaviors. This study examines individual factors that lead to different types of continued substance use while on probation, among a group of people participating in a randomized clinical trial.

## Methods

Substance-using probationers (N =194) completed baseline and 2-month follow-up interviews. Probationers averaged 35 years-old and were predominately male (69%) and non-white (79%). Measures included demographics (e.g., age), treatment/use experience (e.g., ASI drug score), psychosocial functioning (e.g., self-esteem), and criminal justice experience (e.g., lifetime arrests). Hard drug use included opiates, cocaine, barbiturates, amphetamines, hallucinogens, and inhalants. Multinomial regression models examined the relationship between these factors and different category of continued substance use: no use (reference category); hard drug user; marijuana or significant alcohol users only.

## Results

Using nested multinomial regression models, researchers determined that the best fit model included age, recent homelessness, consequences of substance use, prior lifetime treatment, age of first illegal drug use, family and peer drug use, type of substance use at baseline, ASI alcohol and drug scores, hostility, criminal cognitions, and drug-testing probation condition. Multinomial regression results revealed that being younger (Exp(*B*) = .94, *p* = .04), recently homeless (Exp(*B*) = .27, *p* = .03), having an increased ASI alcohol score (Exp(*B*) = 66.43, *p* = .01), or having no drug-testing requirement (Exp(*B*) = 4.09), *p* = .01) were more likely to have continued marijuana or significant alcohol use. Probationers who reported more consequences from substance use (Exp(*B*) = 1.06, *p* = .02), not being an alcohol/marijuana user only at baseline (Exp(*B*) = .22, *p* = .03), having an increased ASI drug score (Exp(*B*) = 209.69, *p* = .02), or having increased criminal cognitions (Exp(*B*) = 5.69, *p* = .05) had increased odds of continued hard drug use.

## Conclusions

During the early period of supervision, there appears to be a limited suppression effect on substance use, given that only 46 percent of the substance-using probationers reported continued use in the 60 days following probation. Identifying probationers who are most at risk for revocation due to continued substance use can assist probation agencies in targeting probationers with the most effective services. These findings support the importance of early screening using risk/needs assessments to determine an individual’s risk of continued substance use and thus potential probation failure.

## Trial registration

NCT01891656

